# Leaching of NMC industrial black mass in the presence of LFP

**DOI:** 10.1038/s41598-024-61569-3

**Published:** 2024-05-11

**Authors:** Yuanmin Zou, Alexander Chernyaev, Muhammad Ossama, Sipi Seisko, Mari Lundström

**Affiliations:** 1 Department of Chemical and Metallurgical Engineering, School of Chemical Engineering, Aalto University, 00076 Aalto, Finland; 2 Metso Outotec Research Center, Kuparitie 10, 28101 Pori, Finland

**Keywords:** Lithium-ion battery, Cathode active material, Reductant, Battery recycling, Leaching, Chemical engineering, Environmental chemistry, Sustainability

## Abstract

This study focuses on the effect of an emerging source of waste, lithium iron phosphate (LFP) cathode materials, on the hydrometallurgical recycling of the currently dominant industrial battery waste that is rich in transition metals (Ni, Co, Mn, and Li). The effects of the dosage of LFP, initial acidity, and timing of LFP reductant addition were investigated in sulfuric acid (H_2_SO_4_) leaching (*t* = 3 h, *T* = 60 °C, *ω* = 300 rpm). The results showed that addition of LFP increased both transition metal extraction and acid consumption. Further, the redox potential was lowered due to the increased presence of Fe^2+^. An initial acidity of 2.0 mol/L H_2_SO_4_ with acid consumption of 1.3 kg H_2_SO_4_/kg black mass provided optimal conditions for achieving a high leaching yield (Co = 100%, Ni = 87.6%, Mn = 91.1%, Li = 100%) and creating process solutions (Co 8.8 g/L, Ni 13.8 g/L, Li 6.7 g/L, Mn 7.6 g/L, P 12.1 g/L) favorable for subsequent hydrometallurgical processing. Additionally, the overall efficiency of H_2_O_2_ decreased due to its decomposition by high concentrations of Fe^2+^ and Mn^2+^ when H_2_O_2_ was added after *t* = 2 h, leading to only a minor increase in final battery metals extraction levels.

## Introduction

As the world seeks to reduce its dependence on fossil fuels and mitigate the effects of climate change, batteries, especially lithium-ion batteries (LIBs), have become a crucial technology for energy storage and transportation in various applications, including portable electronics, electric vehicles (EVs), and renewable energy storage systems^1–3^. Since the manufacturing and use of LIBs have increased significantly, the number of disposed LIBs will increase in the near future, thus the recycling of lithium-ion batteries has attracted significant attention during recent years. The potential of recovering valuable materials from spent batteries is not only economically attractive but also essential for environmental sustainability. However, it should be noted that, currently, full recovery of battery metals is not cost-effective due to the complex structure of LIBs^4^.

A LIB cell consists of anodic and cathodic active materials, electrolyte, separator, current collectors, and packaging components. The dominant battery chemistries in EVs rely on NMC (lithium nickel manganese cobalt oxides) chemistry (LiNi_x_Mn_y_Co_1−x−y_O_2_) with high energy density and better power performance; however, the use of LFP (LiFePO_4_), is increasing rapidly. In 2022, NMC accounted for 60% of market share whereas LFP had a 30% share. The fraction of LFP has increased substantially compared to its 6% share in 2020^5^. Furthermore, LFP is forecast to grow to 47% by 2026 according to ARK Investment Management LLC^6^. LFP batteries have won over the market with the claimed advantages of environment friendliness (reducing toxicity by eliminating the need for nickel (Ni), cobalt (Co), and manganese (Mn), thus minimizing reliance on their supply chains), fire safety (less heat produced), stability (structurally more stable, less capacity loss), and thermal runaway prevention^7–9^. Currently, 90% of LFP batteries are produced by Chinese manufacturers such as BYD and CATL^10^. In addition, major EV manufacturers like Ford, Tesla, Rivian, Mercedes-Benz, and Hyundai have announced plans to shift battery production from other technologies to LFP^11^. However, one of the key challenges in LFP is the lack of established efficient recycling processes and low recycling rates for various elements. For instance, Europe currently lacks industrial facilities for LFP battery recycling.

Recently, the European Battery regulation was published with the aim of recovering 95% of Co, Ni, and 80% of lithium (Li) by 2031^12^. This further motivates the development of effective LIB recycling processes specifically for cathode materials^13^. No requirements were set for Mn, phosphorus (P), aluminum (Al), iron (Fe), fluorine (F), or graphite and thus, in the near future, these elements will still be considered in the industry as impurities rather than recovered products. However, the development of effective leaching and recovery strategies is imperative and can further pave the way toward the circulation and utilization of these currently overlooked elements.

Generally, the recycling of spent LIBs involves firstly discharge, mechanical pre-treatments, optionally pyrolysis for organics removal, sorting, and other pre-processing stages to obtain a sieved mixture of cathode powders and anode graphite, namely black mass(BM). BM is further processed when transition metals are recovered. Ni, Co, and copper (Cu) are currently recovered well from BM^14^, while Li, Mn, and graphite have only recently been considered as potential elements for recovery^15^. In contrast, elements like F, Fe, P, and Al are still dominantly viewed as impurities in LIB recycling procedures due to their low value.

Battery recycling can be enabled by solely hydrometallurgical methods, although usually in state-of-the-art processes they are used in combination with pyrometallurgical methods^4^. In hydrometallurgical recycling, leaching is typically conducted in mineral acids^16,17^ at relatively high acidity (< pH 1) to ensure that all metals remain in solution. However, Porvali et al.^18^ also showed that efficient metals extraction can be achieved even in low acid leaching (*pH* = 1.89) near room temperature (*T* = 30 °C), thus high acidity and temperatures are not always needed for high levels of metals extraction, despite under the conditions conventionally used. The leaching process is a pivotal step in recovering valuable metals from black mass. The leaching of transition metal oxides is electrochemical in nature and requires a reductant (Eq. 1)^19,20^:

1 $$\begin{gathered} {\text{6LiNi}}_{{1/3}} {\text{Mn}}_{{1/3}} {\text{Co}}_{{1/3}} {\text{O}}_{{2}} {\text{ + 18H}}^{ + } {\text{ + 3H}}_{{2}} {\text{O}}_{{2}} { } \to {\text{ 6Li}}^{ + } {\text{ + 2Ni}}^{{2 + }} {\text{ + 2Mn}}^{{2 + }} {\text{ + 2Co}}^{{2 + }} {\text{ + 3O}}_{{2}} {\text{ + 12H}}_{{2}} {\text{O,}} \hfill \\ \Delta {\text{G }} = \, - {1}0{78}.{\text{6 kJ}}/{\text{mol}} \hfill \\ \end{gathered}$$

The conventional reductant used in state-of-the-art processing is H_2_O_2_^21–25^, but several alternative reductants such as Cu^26,27^, Fe^28,29^, glucose^30,31^, ascorbic acid^32^, and NaHSO_3_^33^ have also been studied. The choice of reductant may play a crucial role in determining the leaching efficiency and selectivity of the process and impact on the pregnant leach solution (PLS) composition as well as the consequent solution purification steps required.

With an increasing amount of LFP waste emerging onto the market^34^, the potential for the synergistic use of LFP waste to provide reductive power for LiCoO_2_(LCO)/NMC battery waste leaching has also been hypothesized, due to the presence of Fe^2+^ in the LFP structure. Jiang et al.^35^, Illés et al.^36^, and Xu et al.^37^ achieved high extraction efficiency for Ni, Co, Mn, and Li during the simultaneous leaching of LCO and LFP or LFP and NMC. In addition, LFP can also provide the solution with an abundant source of phosphate ions (PO_4_^3−^), which brings an additional element into the system and may play a substantial role in the subsequent precipitation process for Al^3+^ and Fe^3+^ removal as phosphates^38,39^.

This article investigates the synergistic leaching of industrial NMC111-dominated black mass in the presence of LFP, and particularly the role of LFP in reducing the transition metal oxides present in state-of-the-art battery waste fractions. The hypothesis is that LFP will convert the metals in the NMC cathode active materials to their divalent states, e.g., Co^3+^ to Co^2+^ and Mn^4+^ to Mn^2+^, which are soluble in the acid solution, as shown in Eq. (2). Divalent iron (Fe^2+^) originating from LFP oxidizes to trivalent iron (Fe^3+^) during the process.

2 $$\begin{gathered} {\text{3Fe}}^{{2 + }} {\text{ + 3LiNi}}_{{1/3}} {\text{Mn}}_{{1/3}} {\text{Co}}_{{1/3}} {\text{O}}_{{2}} {\text{ + 12H}}^{ + } \to {\text{ 3Li}}^{ + } {\text{ + Ni}}^{{2 + }} {\text{ + Mn}}^{{2 + }} {\text{ + Co}}^{{2 + }} {\text{ + 3Fe}}^{{3 + }} {\text{ + 6H}}_{{2}} {\text{O,}} \hfill \\ \Delta {\text{G }} = - 51{1}{\text{.8 kJ/mol}} \hfill \\ \end{gathered}$$

Although a previous study^37^ has explored leaching of mixed spent LFP and NMC532, the suggested processing approach necessitated use of higher temperature (90 °C) and the use of additional Fe^3+^ in high concentration (240 g/L). In contrast, the current study operates at lower temperature (60 °C) and aims to eliminate the need for supplementary Fe^3+^ in the hydrometallurgical treatment of industrial NMC111-dominated black mass. This method aligns with industrial practices, offers enhanced operability, and may reduce the economic costs related to addition of external chemicals. Furthermore, adopting this method yields PO_4_^3−^ in the pregnant leach solution, facilitating subsequent purification processes^39^.

The goal of this paper is to discuss the potential of LFP in the realm of black mass recycling to support the advancement of sustainable and economically sound methods for reclaiming valuable metals, not only from state-of-the-art lithium-ion battery waste, but also from future LFP-dominated waste fractions. This, in turn, will contribute to the establishment of a cleaner and more resource-efficient energy storage value chain.

## Materials and methods

### Raw materials

The synthetic active materials used in the initial leaching experiments were synthetic LFP powder (particle size: 1.5 µm (D50), MSE Supplies) and NMC111 powder (particle size: < 0.5 µm(D50), MSE Supplies). The morphology of the above-mentioned NMC111 materials was investigated by XRD and SEM in our previous work^40^, while the XRD diffractogram of synthetic LFP presented in Supplementary Fig. S1 indicates that there was only LiFePO_4_ phase in the material. Table 1 shows the chemical composition of the synthetic NMC111 and LFP. According to Eq. (2), 1 mol of LiFePO_4_ possesses 1 mol of Fe^2+^, and 1 mol of Fe^2+^ will reduce 1 mol of NMC, therefore theoretically 1 mol of LFP is needed to reduce and dissolve 1 mol of NMC. However, it is also known that NMCs also dissolve partially in acid solutions in the absence of additional reductants^41,42^, hence decreasing this ratio slightly in practice.

**Table 1:** Bulk metallic composition of synthetic materials used (wt.%).

	Ni	Mn	Co	Li	P	Fe
NMC111	23.0	16.6	20.5	7.8	–	–
LFP	–	–	–	4.4	19.6	35.4

The industrial BM employed in this study consisted of NMC metal with trace impurities such as F, silicon (Si), and P. The particle size and morphology were observed using a scanning electron microscope (SEM, MIRA 3, Tescan, Czech Republic) equipped with an UltraDry Silicon Drift energy-dispersive X-ray spectrometer (EDS) and utilizing NSS microanalysis software (Thermo Fisher Scientific, USA). To investigate the surface morphology and characteristics of the active material, samples were affixed to carbon tape. The BM was also studied by X-ray diffraction analysis using an X’Pert Pro MPD Powder instrument (USA), equipped with a PIXcel1D detector and a Co Kα source. The XRD apparatus operated at 40 kV and 40 mA, utilizing an Fe beta filter without a monochromator. The chemical composition was quantified through a two-step process: first, complete dissolution in aqua regia, followed by elemental analysis using atomic absorption spectroscopy (AAS) with a Thermo Fisher ICE 3000 instrument, operated with an air-acetylene flame. Al and P were measured using inductively coupled plasma-optical emission spectrometry (ICP-OES, Agilent 5900 SVDV). Additionally, the determination of fluoride content was carried out using the DIN 51723: 2002-06 method (Measurlabs, Helsinki). The characterization results are presented in the Results chapter.

The lixiviants were prepared from sulfuric acid (VWR Chemicals, 95%) to the desired concentration (0.3 M, 0.5 M, 1.0 M, and 2 M H_2_SO_4_). The accuracy of the concentration was verified through titration, using a standardized 2 M NaOH solution (Merck, Titripur). H_2_O_2_ (VWR Chemicals, 50%, GPR Rectapur) was added in some tests.

### Leaching experiments procedure

First, the fundamentals of the leaching behavior of LFP and NMC were studied with synthetic raw materials in order to avoid any anomalies due to the impact of impurities (E1–E10). A 500 mL round-bottomed glass reactor equipped with a water jacket for heating (*T* = 30 °C) was used. The solution volume was set to 400 mL and agitation was maintained at 300 rpm with a four-blade stirrer. The duration of the experiment was 2 h, and samples were collected at specified time intervals (at 10 min, 15 min, 30 min, 60 min, and 120 min). The redox potential was monitored from within the reactor vessel using an oxidation–reduction potential (ORP) electrode (Pt vs. Ag/AgCl in a 3 M KCl salt bridge, Mettler Toledo, USA).

Table 2 shows the detailed information about the exploratory leaching (E1–E10). Experiments E1–E3 were conducted to investigate the influence of acidity on the solubility of synthetic LFP only, whereas in E4 the potential impact of H_2_O_2_ on LFP leaching was also explored. In experiments E5–E8 the synergistic impact of LFP and NMC leaching was studied, with different molar ratios of LFP/NMC (0.3–0.9). In addition, the impact of acidity on synergistic leaching was observed in E8–E10. The extraction *Y*_Me_ (in %) in the solution was calculated using Eq. (3):

3 $${\text{Y}}_{{\text{Me }}} { = }\frac{{{\text{C}}_{{{\text{Me}}}} \cdot {\text{V}}_{{\text{L}}} }}{{{\text{x}}_{{0}} \cdot {\text{m}}_{{0}} }} \cdot {\text{100}}\%,$$

where *C*_Me_ is the concentration of metal (g/L) in solution, *V*_L_ is the volume of the leach solution (L), *x*_*0*_ is the fraction of metal in the concentrate (%), and *m*_*0*_ is the initial weight of the concentrate introduced to the leaching reactor (g).

**Table 2:** Exploratory leaching experiments (E1–E10) of synthetic cathode materials (*T* = 30 °C, *S/L* = 31–78 g/L, *t* = 2 h, *V* = 400 mL).

Test	Acidity/M	NMC111/g	LFP added/g	Molar ratio of (LFP/NMC)	Molar ratio of (LFP/(sum Ni + Mn + Co))	Time of 1 vol.% H_2_O_2_ addition/min
E1	2	0	12.5	–	–	–
E2	0.5	0	12.5	–	–	–
E3	0.3	0	12.5	–	–	–
E4	2	0	18.7	–	–	30
E5	2	12.7	0	–	0	–
E6	2	12.7	6.2	0.3	0.3	–
E7	2	12.7	12.5	0.6	0.6	–
E8	2	12.7	18.7	0.9	0.9	–
E9	1	12.7	18.7	0.9	0.9	–
E10	0.5	12.7	18.7	0.9	0.9	–

In the second leaching series, industrial NMC111 battery waste (BM) was used. In order to limit anomalies due to impurities, synthetic LFP was used as the synergistic reductant, Table 3. Leaching was conducted in a 1 L round-bottomed glass reactor equipped with a four-blade stirrer (300 rpm) with a water jacket. The solution volume was set at 500 mL. The experiment duration was 3 h, and samples were collected at specified time intervals (at 5 min, 15 min, 30 min, 45 min, 60 min, 120 min, and 180 min). The investigated S/L ratio varied from 100 to 156.5 (g/L). LFP was introduced as a potential reductant to facilitate synergistic leaching of NMC and LFP. T1–T5 investigated the impact of the reductant concentration (LFP) on transition metal extraction. T5–T6 and T7–T8 were conducted to study how the timing of LFP addition (*t* = 0 or 1 h) in concentrations of 1 M and 2 M sulfuric acid. Finally, the impact of H_2_O_2_ as additional reductant at *t* = 2 h was observed (T9–T10). Redox potential measurements were taken using an ORP electrode (Pt vs. Ag/AgCl in 3 M KCl salt bridge, Mettler Toledo, USA). All the leaching experiments were conducted in a dedicated LIB LAB space (Aalto University), employed for the safe handling of industrial BM. The extraction *Y*_Me_ (in %) in the solution was calculated using Eq. (3).

**Table 3:** BM leaching experimental series (*T* = 60 °C, *S/L* = 100–156 g/L, *t* = 3 h, *V* = 500 mL).

Test	Acidity/M	Black mass/g	LFP added/g	Molar ratio of (LFP/(sum Ni + Mn + Co))	Time of LFP addition/min	Time of 2 vol.% H_2_O_2_ addition/min
T1	2	50	0	0.000	0	–
T2	2	50	3.5	0.1	0	–
T3	2	50	7.1	0.2	0	–
T4	2	50	14.1	0.3	0	–
T5	2	50	28.3	0.6	0	–
T6	2	50	28.3	0.6	60	–
T7	1	50	28.3	0.6	60	–
T8	1	50	28.3	0.6	0	–
T9	2	50	28.3	0.6	0	120
T10	1.5	50	28.3	0.6	0	120

It is worth noting that the lithium leaching efficiency in both E1–E10 and T1–T10 refers to the mixture of NMC and LFP in systems where both battery chemistries were studied. In addition, it should be noted that the Gibbs free energy change values (∆G of NMC111, LFP, Fe(H_2_PO_4_)_2_, H_2_O_2_, and O_2_) estimated by HSC 10 Chemistry (software version 10.0.6.4, Metso Outotec)^43^ at 25 °C were used in the ∆G calculation of all equations in the current study since there were no exact thermodynamical data values in the database of the HSC 10 Chem software, while the ∆G of the other chemical species were taken from that database.

## Results

### Interaction of NMC with LFP (synthetic materials)

In order to study the potential for synergistic leaching of future battery waste (LFP) with the currently dominant battery waste chemistries (NMC), cathode active materials and their interactions were first studied with synthetic materials.

**Figure 1:**
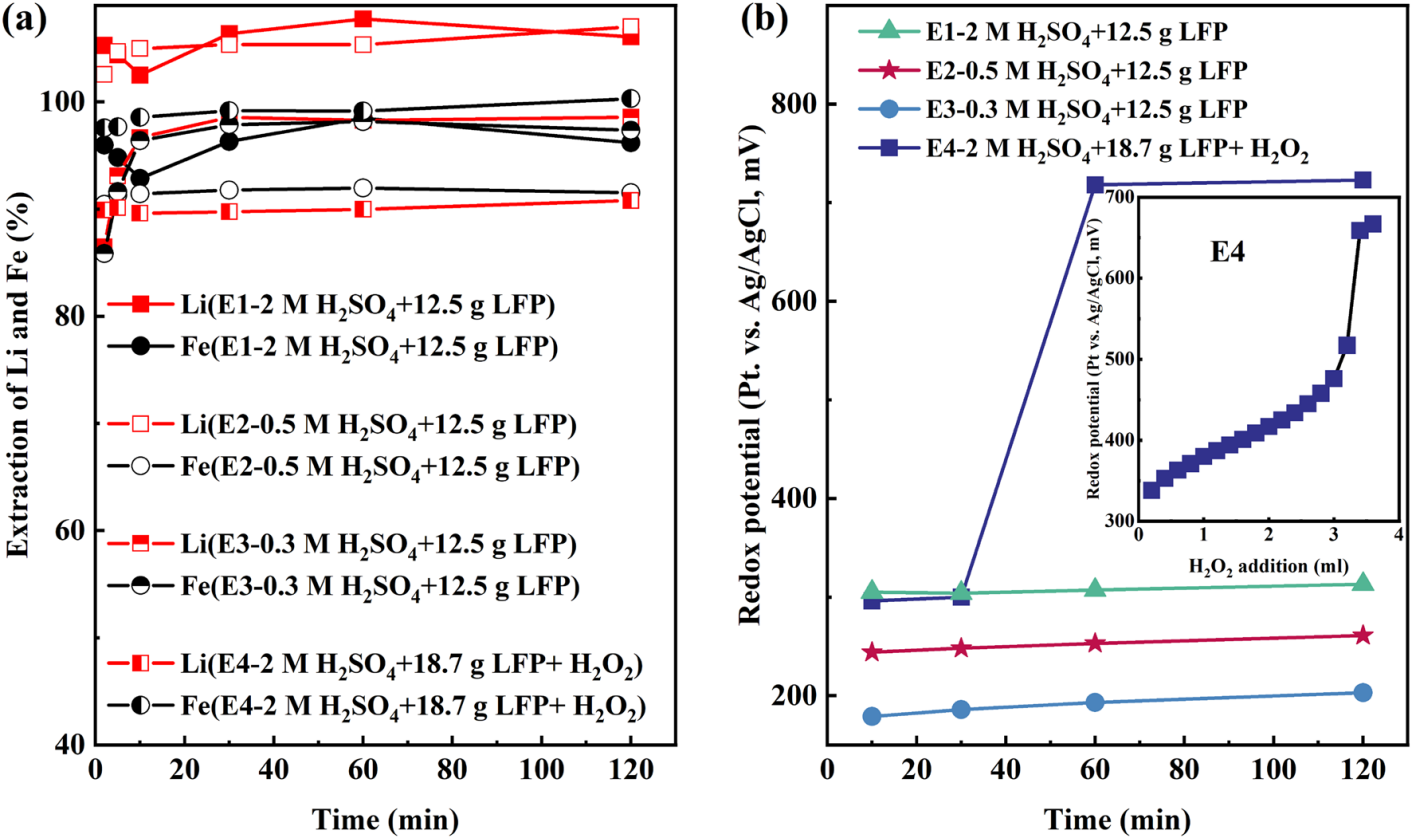
(**a**) Extraction of Li and Fe in E1–E4 and (**b**) redox potential of E1–E4.

First, synthetic LFP alone was leached in sulfuric acid solutions, see Fig. 1a. LFP exhibited high dissolution in the sulfuric acid leaching system with a maximum of 96% of LFP dissolved in 2 M H_2_SO_4_ lixiviant. H_2_O_2_ did not improve LFP dissolution but was consumed in the oxidation of Fe^2+^ to Fe^3+^, indicated by a gradual rise in redox potential from 335 to 476 mV vs. Ag/AgCl as H_2_O_2_ was added (from 0 to 3 mL). Subsequently, the redox potential experienced a pronounced surge, spiking from 476 to 718 mV vs. Ag/AgCl with the introduction of the remaining 1 mL of H_2_O_2_ in the interval between 30 and 60 min when combining the results shown in Fig. 1b. The results suggest that LFP is acid soluble and that high levels of extraction from this cathode material can be achieved without externally added oxidants or reductants, whereas most of the iron remains in the form of Fe^2+^.The acidity levels (Supplementary Fig. S2a) decreased from 2 M to 1.9 M in E1, from 1 M to 0.4 M in E2, and from 0.5 M to 0.1 M in E3, indicating that the dissolution of LFP in sulfuric acid to form PO_4_^3−^, which could consume H^+^ form HPO_4_^2−^, H_2_PO_4_^−^, and H_3_PO_4(aq)_ as shown in Eq. (4):

4 $$\begin{gathered} {\text{2LiFePO}}_{{4}} {\text{ + 3H}}_{{2}} {\text{SO}}_{{4}} {\text{ = 2FeSO}}_{{4}} {\text{ + Li}}_{{2}} {\text{SO}}_{{4}} {\text{ + 2H}}_{{3}} {\text{PO}}_{{4}} {, } \hfill \\ \Delta {\text{G}} = - 6{7}{\text{.4 kJ/mol}} \hfill \\ \end{gathered}$$

**Figure 2:**
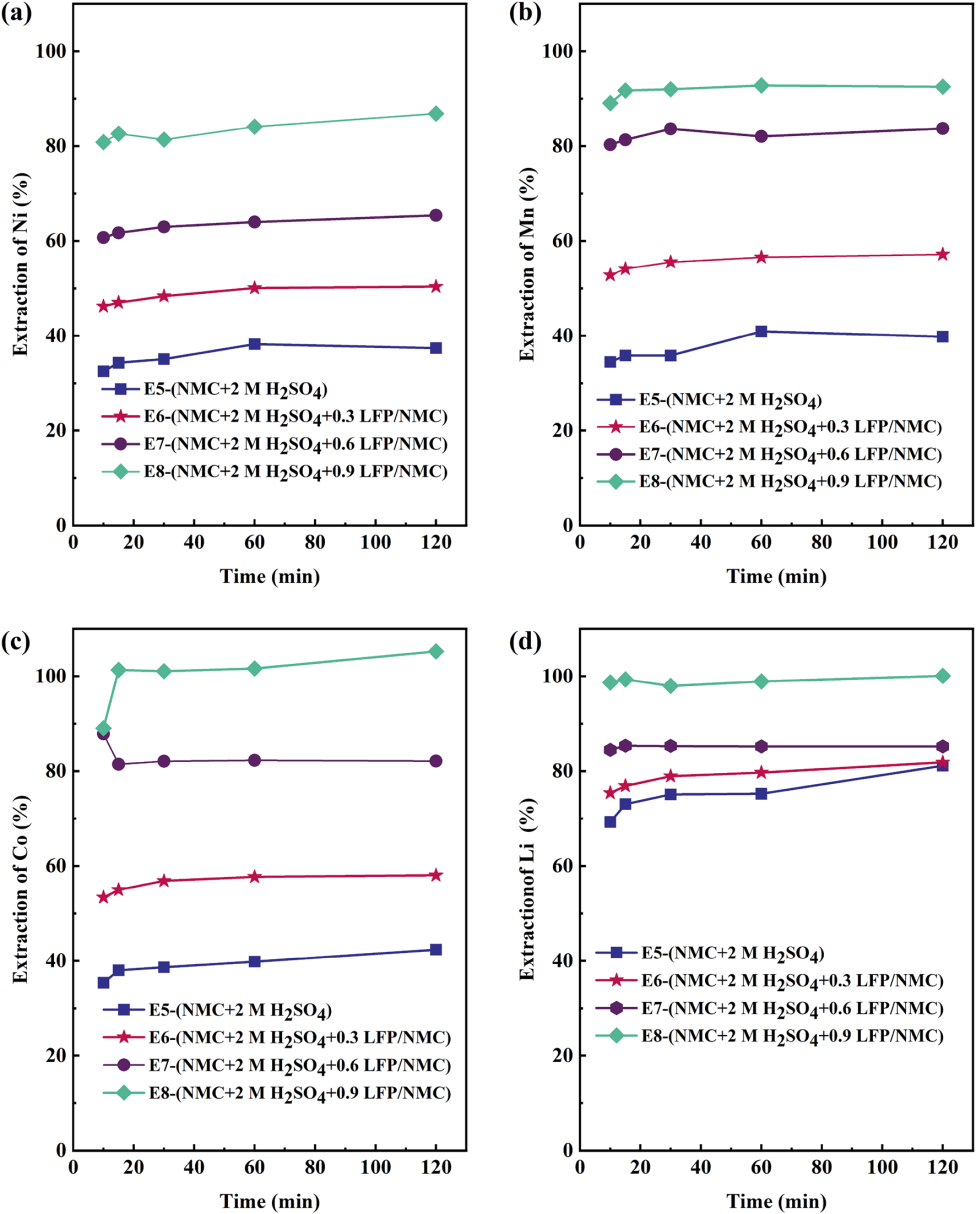
Metal extraction of NMC111 leaching for (**a**) Ni; (**b**) Mn; (**c**) Co; (**d**) Li.

Secondly, synthetic LFP was leached together with NMC111, with the increased LFP addition clearly raising the extraction of target metals (Ni, Co, Mn, and Li), see Fig. 2. In the absence of LFP (E1), the Ni leaching yield was 37.4%, while Ni extraction increased to 86.9% at the highest (E8, LFP/NMC ratio of 0.9). Similarly, the leaching yield for Co increased from 42.4% to full extraction, that of Mn from 39.8% to 92.5%, and that of Li from 81.1% to full extraction. Due to the increased addition of LFP, the final Li concentrations increased as well, to 2.0 g/L in E5, 3.2 g/L in E6, 4.0 g/L in E7, and 4.6 g/L in E8 (Supplementary Fig. S3a). The results suggest that addition of LFP–providing the system with dissolved Fe^2+^ as reductant^26^—can improve synthetic NMC leaching almost linearly within the studied range (LFP/NMC ratio of 0–0.9). Furthermore, the leaching response of all transition metals (Mn, Ni, and Co) was found to be quite similar during this synergistic leaching. The acidity levels (Supplementary Fig. S2b) decreased from 2 M to 1.6 M in E5, to 1.7 M in E6, to 1.4 M in both E7and E8, indicating that with further addition of LFP, more acid is consumed in reactions such as Eq. (5):

5 $$\begin{aligned} & {\text{6LiNi}}_{{1/3}} {\text{Mn}}_{{1/3}} {\text{Co}}_{{1/3}} {\text{O}}_{{2}} {\text{ + 12H}}_{{2}} {\text{SO}}_{{4}} {\text{ + 6FeSO}}_{{4}} { } \to {\text{ 2NiSO}}_{{4}} {\text{ + 2MnSO}}_{{4}} {\text{ + 2CoSO}}_{{4}} {\text{ + 3Li}}_{{2}} {\text{SO}}_{{4}} \\ & \quad {\text{ + 3Fe}}_{{2}} {\text{(SO}}_{{4}} {)}_{{3}} {\text{ + 12H}}_{{2}} {\text{O, }}\quad \Delta {\text{G = - 1509}}{\text{.9 kJ/mol}} \\ \end{aligned}$$

**Figure 3:**
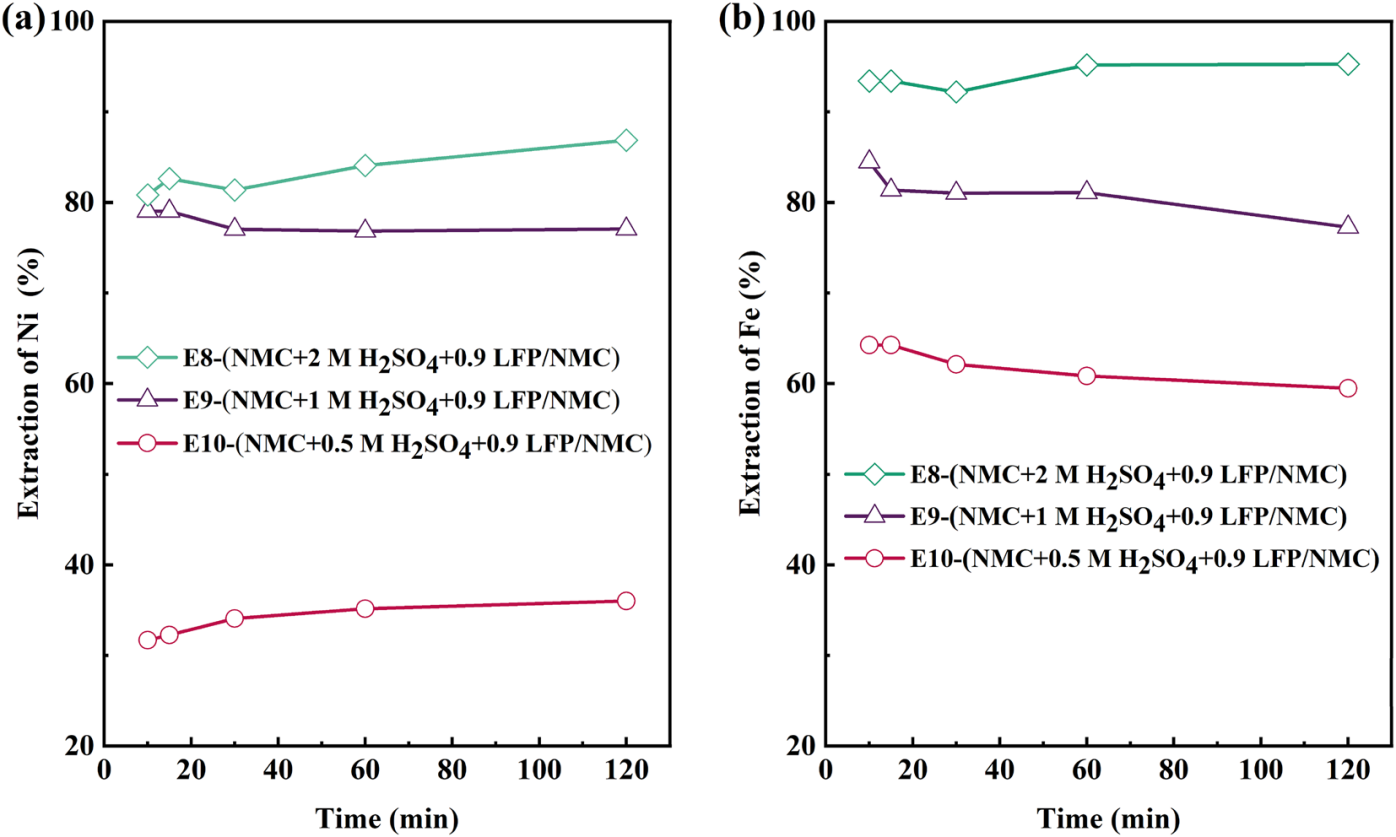
(**a**) Ni and (**b**) Fe leaching yield at different H_2_SO_4_ concentrations.

The influence of acid concentration on the leaching performance of NMC111 was further examined by varying the initial H_2_SO_4_ concentration (2 M, 1 M, and 0.5 M) while maintaining a constant molar ratio of 0.9 between LFP and NMC111. The outcomes, shown in Fig. 3a, reveal that the extraction of Ni was maximized in the 2 M leaching system (E8) and minimized in the 0.5 M leaching system (E10), where the final pH value remained at 0.9, leading to no precipitation related to Ni. Co and Mn exhibited similar behavior to Ni, with the highest leaching efficiency observed in the 2 M acid system (E8) and the lowest leaching efficiency in the 0.5 M acid system (E10) (Figures S4 a and b), where the leaching yields were only 65.4% for Ni, 40.2% for Co, and 41.9% for Mn (E10), when the initial acidity was 0.5 M, resulting in the lower dissolution of LFP, hence less reductant (Fe^2+^) available to reduce NMC111 leading to lower leaching efficiency.

For lithium, full extraction could be obtained in the 2 M acid system (Supplementary Fig. S4c), with 92.2% dissolved in the 1.0 M acid system and only 73.7% dissolved in the 0.5 M acid system. The lithium concentration was 4.6 g/L in E8, and slightly lower in E9 (4.2 g/L) and E10 (3.4 g/L) (Supplementary Fig. S3a). The lower Li concentrations in E9 and E10 may be explained by the lower acidity used in these experiments, the lower proton concentration weakening the lithium deintercalation in the NMC111 particles and limiting the surface-controlled dissolution^41^.

According to Eqs. (4) and (5), the holistic leaching reaction can be suggested as Eq. (6):

6 $${\text{6LiNi}}_{{1/3}} {\text{Mn}}_{{1/3}} {\text{Co}}_{{1/3}} {\text{O}}_{{2}} {\text{ + 21H}}_{{2}} {\text{SO}}_{{4}} {\text{ + 6LiFePO}}_{{4}} { } \to {\text{ 2NiSO}}_{{4}} {\text{ + 2MnSO}}_{{4}} {\text{ + 2CoSO}}_{{4}} {\text{ + 6Li}}_{{2}} {\text{SO}}_{{4}} {\text{ + 3Fe}}_{{2}} {\text{(SO}}_{{4}} {)}_{{3}} {\text{ + 6H}}_{{3}} {\text{PO}}_{{4}} {\text{ + 12H}}_{{2}} {\text{O, }}\quad \Delta {\text{G }} = - 1{712}{\text{.0 kJ/mol}}$$

Additionally, iron can form aqueous phosphate species in the current system such as FeH_2_PO_4_^2+^, FePO_4(aq)_, FeH_2_PO_4_^+^, as well as Fe_3_(PO_4_)_2(aq)_^44^ (Eq. 7). However, at high acidity (pH < 1) any aqueous divalent iron phosphate species—if present—are hypothesized also acting as reductant, resulting in net reactions similar to that of Eq. (6).

7 $${\text{2LiFePO}}_{{4}} {\text{ + 2H}}_{{2}} {\text{SO}}_{{4}} {\text{ = FeSO}}_{{4}} {\text{ + Li}}_{{2}} {\text{SO}}_{{4}} {\text{ + Fe(H}}_{{2}} {\text{PO}}_{{4}} {)}_{{2}} \quad \Delta {\text{G }} = - 63.{\text{0 kJ/mol}}$$

Experimental results show that higher acidity as well as increased LFP addition can facilitate higher concentrations of Fe^2+^ to the solution, boosting enhanced reduction of NMC111. In addition, the solubility of iron as well as transition metals may limit the process at elevated pHs. The leaching of LFP produces H_3_PO_4_ from H_2_SO_4_ (Eq. 4) instead of water, and therefore iron phosphate remains in aqueous form at higher acidity, as shown in Pourbaix diagram in the study of Jing et al.^45^. In the experimental series conducted for pure materials (E5–E8) at 2 M H_2_SO_4_ (*pH* = -0.6), this was experimentally confirmed as no iron was observed to precipitate, whereas Fe solubility was limited in the lower acidity experiments (E9–E10) (Fig. 3b). In order to maximize the impact of Fe^2+^ as reductant and to minimize the risk of iron precipitate formation, the 2 M acid was selected as the lixiviant for the leaching of industrial black mass, experimental series T.

### Industrial black mass characterization

Industrial BM varies considerably in composition and chemistry; consequently, the received material was analyzed in detail. Table 4 shows the composition of the industrial BM, and the synthetic material compositions are shown in Table 1. The Ni content in the BM is nearly twice that of Mn and Co. Fe, in contrast, constitutes a minor fraction, making up only 0.1 percent of the black mass. The Al and Cu content are similar, representing 2.3–2.4% each. The fluoride content in the BM was ca. 1%, whereas P was also present (9.8%) in the studied industrial battery waste fraction.

**Table 4:** Bulk metallic composition of materials used (wt.%).

	Ni	Mn	Co	Li	P	Fe	Cu	Al	F
Black mass	15.8	8.4	8.7	4.1	9.8	0.1	2.3	2.4	1.0

**Figure 4:**
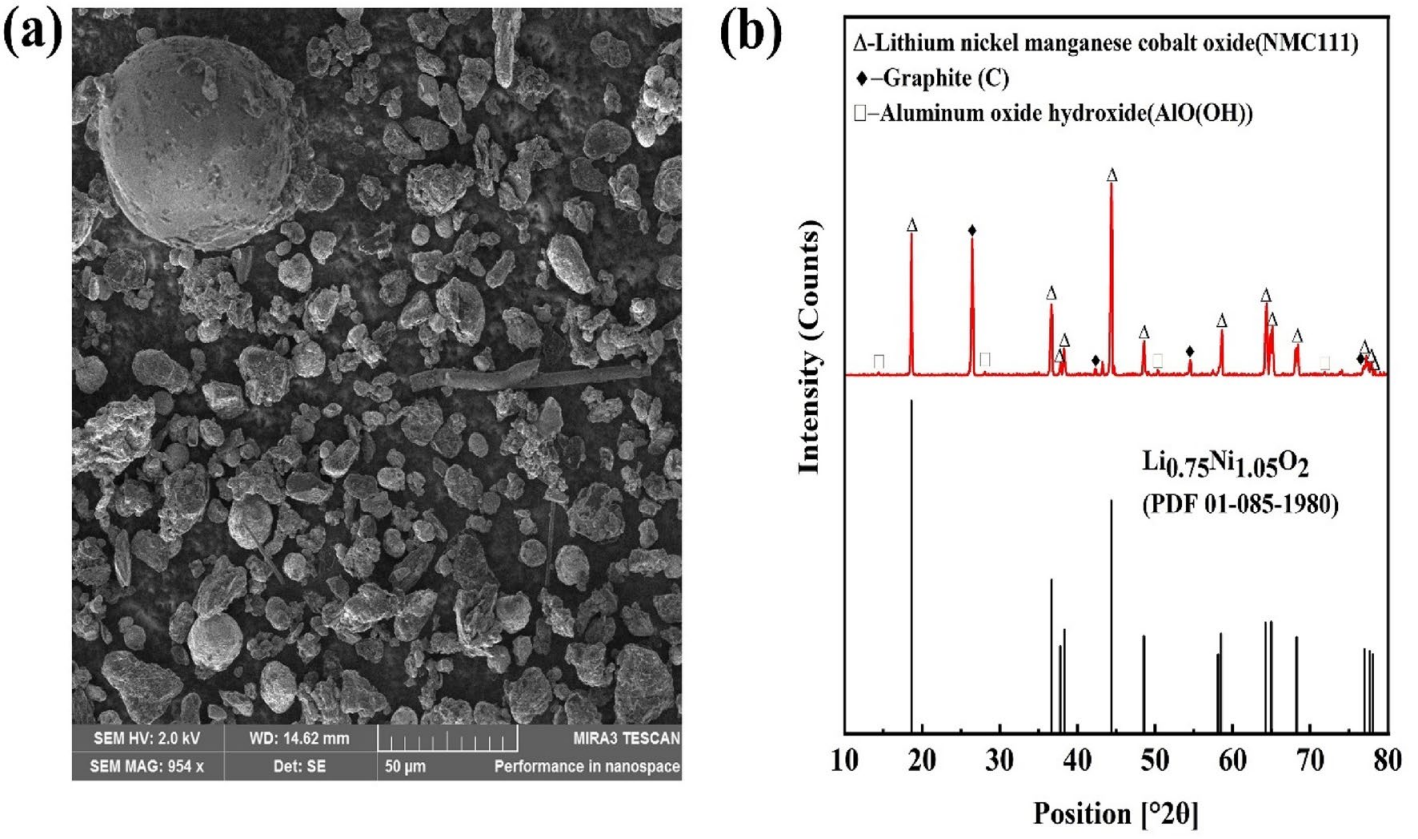
(**a**) SEM micrograph and (**b**) XRD diffractogram of investigated black mass.

The SEM micrograph of BM shown in Fig. 4a reveals a highly heterogeneous morphology with distinct variations in both particle size and shape, compared with typical NMC material, which has spherical particles of uniform size^46,47^. The BM particles exhibit remarkable diversity, displaying forms such as spherical, branched, ellipsoidal, and other irregular shapes. The size differences between particles range from small (2 µm) individual spheres to larger particles (95 µm), which appear to be agglomerates formed by the aggregation of numerous smaller particles. The surface texture of these particles is characterized by a non-smooth, irregular, and bumpy appearance, further underscoring the complex nature of this BM morphology. As Fig. 4b illustrates, the peaks of the obtained XRD pattern match the graphite (C), boehmite (AlO(OH)), and NMC111 peak standards as well as a separate lithium nickel oxide (Li_0.75_Ni_1.05_O_2_). The combined results of XRD and AAS analysis suggest that this black mass may consist mainly of two different battery chemistries.

### Reductive leaching of industrial black mass with LFP addition

The leaching of industrial black mass focused on studying the impact of the leaching parameters and the positive effect of the LFP waste fraction on metal extraction. This section provides an analysis and discussion of how different acid concentrations, reductant mass, and the timing of its addition impact the leaching process.

#### Impact of LFP addition

**Figure 5:**
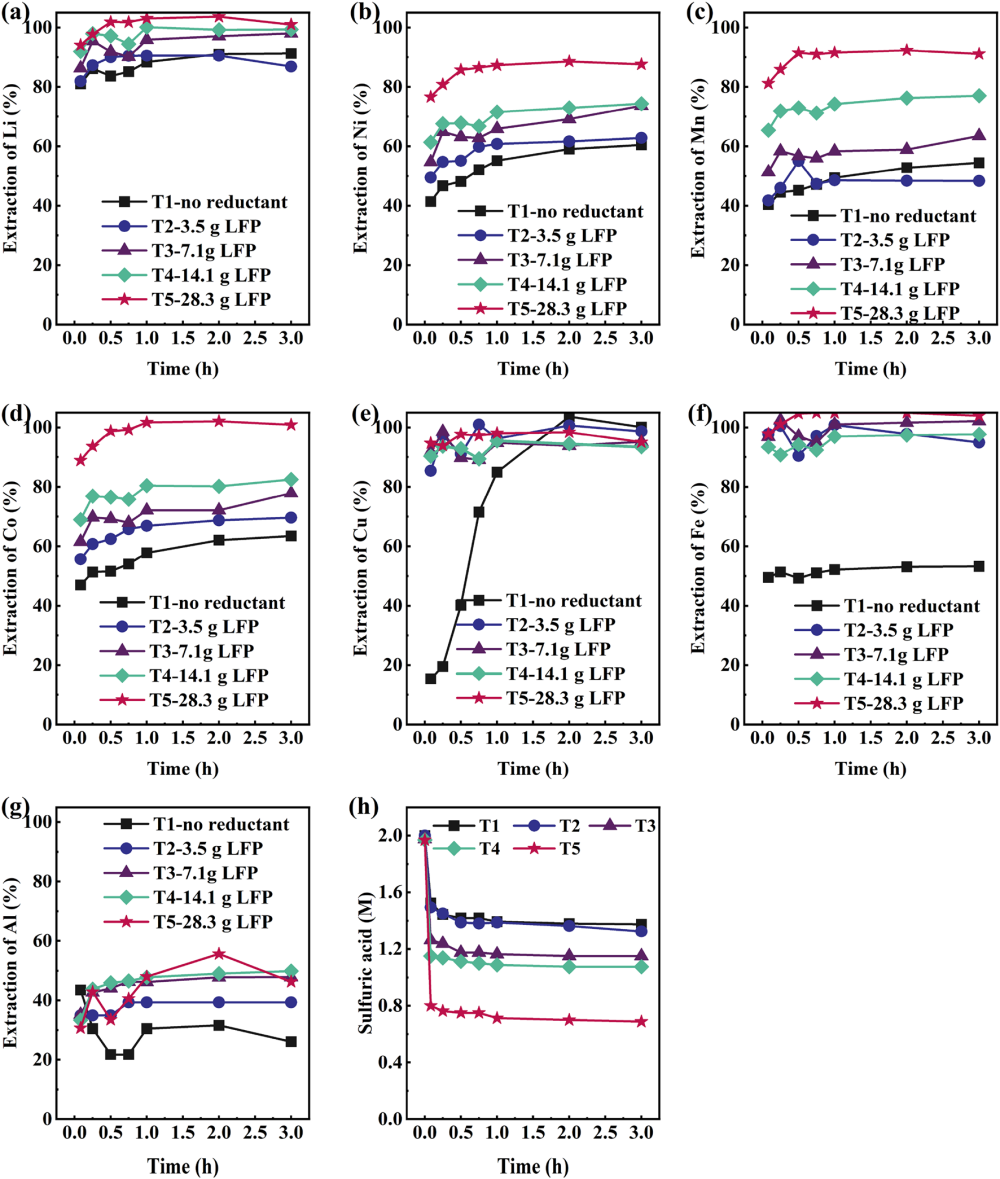
Leaching yield from industrial BM for (**a**) Li; (**b**) Ni; (**c**) Mn; (**d**) Co; (**e**) Cu; (**f**) Fe; (**g**) Al; and (**g**) Acidity of samples (Experiments T1–T5).

Similar to the synthetic leaching experiments, the addition of LFP during industrial NMC BM leaching led to an increase in the extraction of target metals, as earlier suggested by Eq. (2), see Fig. 5. However, leaching of industrial NMC BM alone (T1)—without LFP addition—resulted in substantially higher target metal extraction levels (60.5%, 54.5%, 63.5%, and 91.3% for Ni, Mn, Co, and Li, respectively), when compared to synthetic NMC111 leaching, where < 40% of transition metals were extracted and < 82% of Li (E5). This can be explained by the presence of the impurities Cu (2.3%) and Al (2.4%) in the industrial raw material, which are also able to act as in-situ reductants for the system^26,40,48^ (Eq. 8). The reductive ability of Cu is confirmed by the 100% Cu leaching in T1, whereas Al reduction efficiency is known to suffer from a passivation tendency^49^, which explains why only 26.1% of Al was leached.

8 $${\text{6LiNi}}_{{1/3}} {\text{Mn}}_{{1/3}} {\text{Co}}_{{1/3}} {\text{O}}_{{2}} {\text{ + 12H}}_{{2}} {\text{SO}}_{{4}} {\text{ + 3Cu }} \to {\text{ 2NiSO}}_{{4}} {\text{ + 2MnSO}}_{{4}} {\text{ + 2CoSO}}_{{4}} {\text{ + 3Li}}_{{2}} {\text{SO}}_{{4}} {\text{ + 3CuSO4 + 12H}}_{{2}} {\text{O }}\quad \Delta {\text{G }} = - 16{52}{\text{.7 kJ/mol}}$$

When LFP is added to a real industrial BM leaching system, not only Cu and Al, but also Fe^2+^ can act as an in-situ reductant (T2–T10). It is worth highlighting that only a very low concentration of iron was originally present in the black mass (Table 4), thus the dominant source of iron entering the system was LFP. After the reduction reaction, such as Eq. (5), trivalent iron can further oxidize the metallic impurities present (Cu, Al), thus regenerating the reductive power of iron back to Fe^2+^. This catalyzing effect is well known in the state-of-the-art battery leaching literature^26^. The objective of LFP addition in the current study was initially to keep the final iron concentrations in experiments T2, T3, T4, and T5 at 2.5 g/L, 5 g/L, 10 g/L, and 20 g/L, respectively. In Fig. 5e, it is apparent that copper indeed underwent rapid oxidation upon the introduction of LFP, due to the Fe^3+^ (formed via Eq. 5) leading to the oxidation of copper to Cu^2+^, and the consequent generation of Fe^2+^. However, by this reaction route, neither the reductive power of LFP nor Cu is lost but can further boost NMC leaching^40^. Thus, also in industrial black mass leaching, the extraction of target metals seems to increase almost linearly with LFP addition.

With the highest investigated LFP addition (T5, 0.6 mol LFP/mol (sum Ni + Mn + Co) ratio, Table 3), as high as 100%, 87.6%, 91.1%, 100%, and 95.0% yields could be achieved for the target elements Li, Ni, Mn, Co, and Cu, respectively. Full extraction could be achieved for lithium and cobalt, while nickel and manganese extractions were close to 90%, increasing by 28%-units and 37%-units when compared to experiment T1. Additionally, Fig. 5f confirms that the amount of iron dissolved solely from the investigated BM was low (0.02 g/L), whereas with LFP addition it increased significantly, up to 20.9 g/L in the case of T5.

Acid consumption was also observed to increase linearly with addition of LFP (Fig. 5h) from T1 to T5. The final acidity of experiment T1 measured 1.4 M, while in the case of T5, it was recorded at 0.7 M. This observation demonstrates that when a greater amount of LFP is added initially—and more of the target metals are dissolved—more acid is consumed (acid consumption (kg H_2_SO_4_/kg BM) was 0.6 in T1, 0.7 in T2, 0.8 in T3, 0.9 in T4, and 1.3 in T5, respectively). This consumption is a result of the increased dissolution of active material, which leads to a greater consumption of hydrogen ions (Eqs. (4) and (5)), consequently reducing the final leach solution acidity. The redox potential in experiments T1–T4 remained high (close to 1000 mV vs. Ag/AgCl at the end of the experiments), reflecting the high oxidation potential of the black mass active materials and high oxidation rate of Fe^2+^ to Fe^3+^. Only in T5 did the redox potential remain below 800 mV vs. Ag/AgCl, most likely due to the high dissolution of oxidative NMC structures as well as a sufficient amount of Fe^2+^ present in the system (Supplementary Fig. S5).

#### Impact of acidity

**Figure 6:**
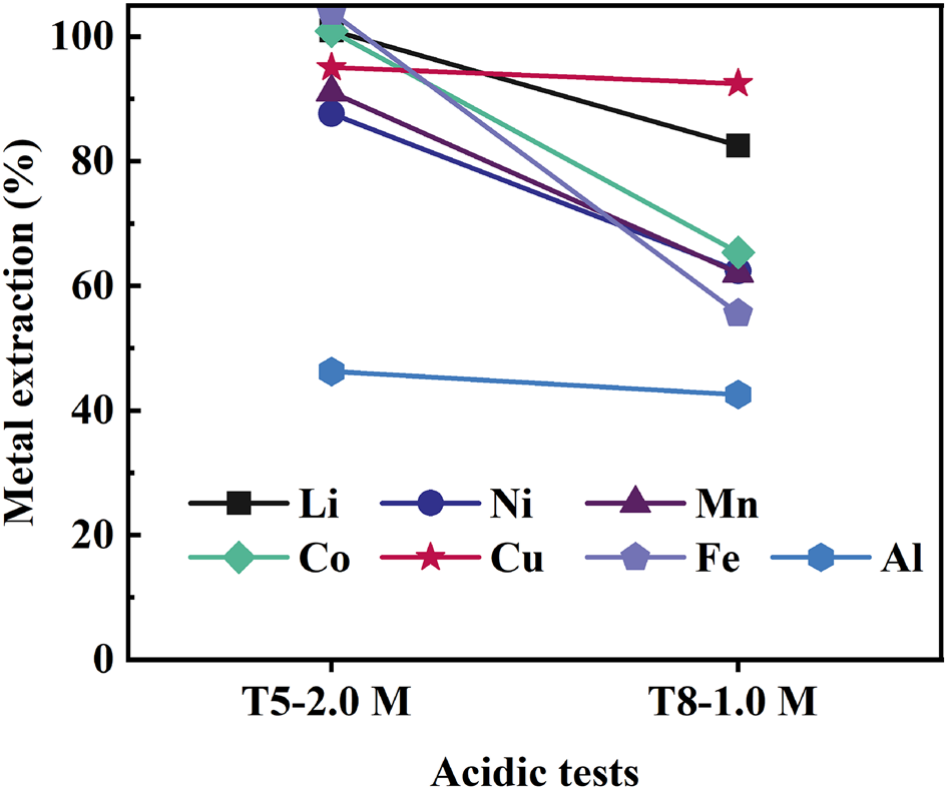
Metal extraction of T5 and T8 with addition of 28.250 g LFP.

In Fig. 6, battery metals leaching yield is presented for two different initial acidities (2 M–T5 and 1 M–T8) with an identical addition of LFP (the molar ratio of LFP/(sum Ni + Co + Mn) kept at 0.6). Similar to the synthetic experiments (E8–E10), also with real industrial black mass, lower acidity (T8, 1.0 M acid) resulted in lower extraction of metals (82.5% Li, 62.4% Ni, 62.0% Mn, 65.3% Co, 92.5% Cu, 55.6% Fe, and 42.5% Al), whereas at 2 M (T5) full leaching was achieved for Li, Co, and Fe, as well as for Ni, Mn, Cu, and Al the values were 87.6%, 91.1%, 95.0%, and 46.3%, respectively. The final acidity in T8 was measured to be 0.15 mol/L (pH = 0.5), indicating that iron phosphate precipitation may not have occurred^50^, but formed species such as FeH_2_PO_4_^2+^, FePO_4(aq)_, FeH_2_PO_4_^+^, and Fe_3_(PO_4_)_2(aq)_. Lower initial acidity led to low solubility of iron and/or precipitation of iron phosphates (55.6%).

#### Impact of timing of addition of LFP reductant and H_2_O_2_

**Figure 7:**
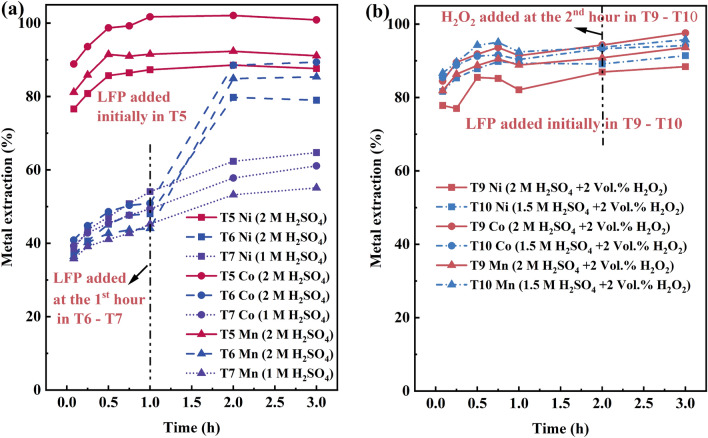
Extraction of Ni, Co, and Mn in (**a**) T5, T6, and T7; (**b**) T9 and T10.

In order to study the impact of LFP, the time of reductant addition was studied. Figure 7a presents a comparison of when LFP was introduced to the system one hour after the start of the experiment (2 M and 1 M lixiviant). First, the metal extraction in T6 and T7 at the time of LFP addition (*t* = 1 h) was nearly identical. Following the introduction of LFP in T6, the extraction percentages for Ni, Co, and Mn increased substantially up to 79.0%, 89.4%, and 85.4%, respectively, while that of lithium was 97%. In contrast, in T7 (at lower acidity) the final yields remained low, 64.7% for Ni, 61.1% for Co, and 55.1% for Mn, as in all of the earlier leaching experiments with low acidity (E9, T8). The final transition metal extractions were still highest if LFP was added immediately (T5) compared to being added after 1 h of leaching. This indicates that the early presence of a high reductant concentration—and also a great amount of catalyzing Fe^2+^/Fe^3+^ as well as phosphates—is beneficial for the system. The presence of a high concentration of reductive components (thermodynamics) may boost black mass leaching kinetics, whereas the positive impact may also be partially related to the formation of aqueous aluminum phosphates in the system, such as AlH_2_PO_4_^2+^ and AlPO_4(aq)_, which may stabilize aluminum in the solution and decrease its passivation tendency. In such cases, more aluminum (impurity in the solution) is available for reduction of active materials^27^, when compared to a pure sulfuric acid system.

Lastly, the addition of H_2_O_2_ to the system was also studied, in experiments T9 and T10. However, this showed only a slight increase in the final target metal leaching yields, nickel extraction increasing 2.5%-units in T9 and 2.3%-units in T10, respectively. Similarly, manganese extraction increased 2.8%-units and 2.1%-units, while cobalt extraction increased 3.3%-units and 0.8%-units, respectively (Fig. 7b). It was worth noting that these shifts in extraction were relatively minor and are not deemed significant. This could potentially be attributed to a reduction in the efficiency of H_2_O_2_, which may decompose due to the high concentrations of Fe^2+^ and Mn^2+^, leading to a decrease in its overall effectiveness.

**Figure 8:**
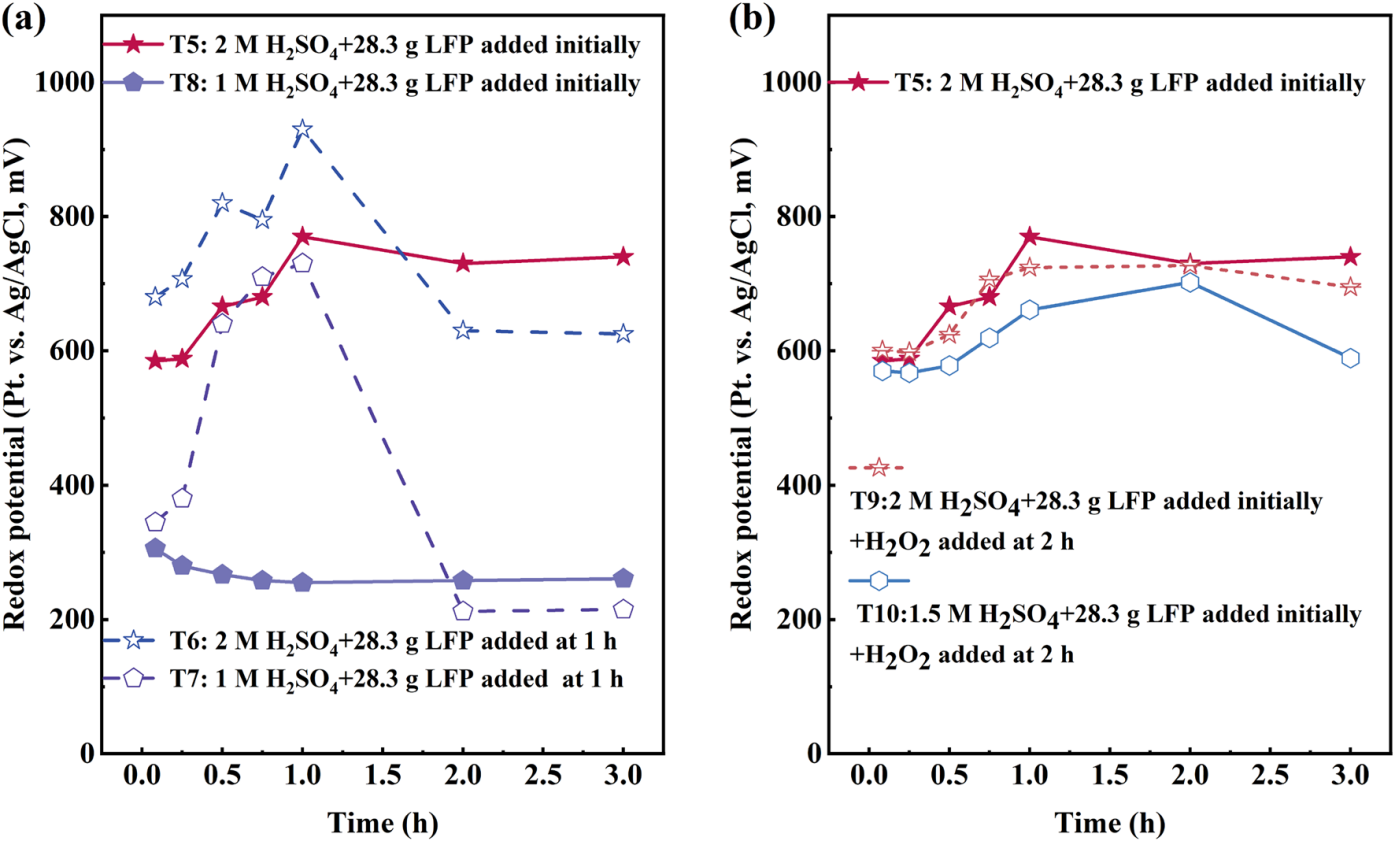
Redox potential of (**a**) T5–T8, (**b**) T5, T9, and T10.

Redox potential was shown to be impacted by the acidity and LFP addition. Higher acidity (T5, T6) provided higher redox potentials in general; however, LFP addition during the experiment (T6, T7, *t* = 1 h) did have a clear decreasing impact on the redox potential, see Fig. 8a. The titrimetric determination of Fe^2+^ using potassium dichromate solution (K_2_Cr_2_O_7_) revealed that there was 74.2% Fe^2+^ in the total Fe content of the final solution in T7 and 71.6% Fe^2+^ in the total Fe content of the final solution in T8. The final acidity for T6 and T7 was 0.7 M and 0.2 M, respectively. This indicates that a significant portion of ferrous iron remained unoxidized into ferric iron in lower acidic conditions, thereby contributing to the low redox potential and metal extraction observed in experiments T7 and T8. Addition of H_2_O_2_ at *t* = 2 h did not have any increasing impact on the solution redox potential, see Fig. 8b.

**Figure 9:**
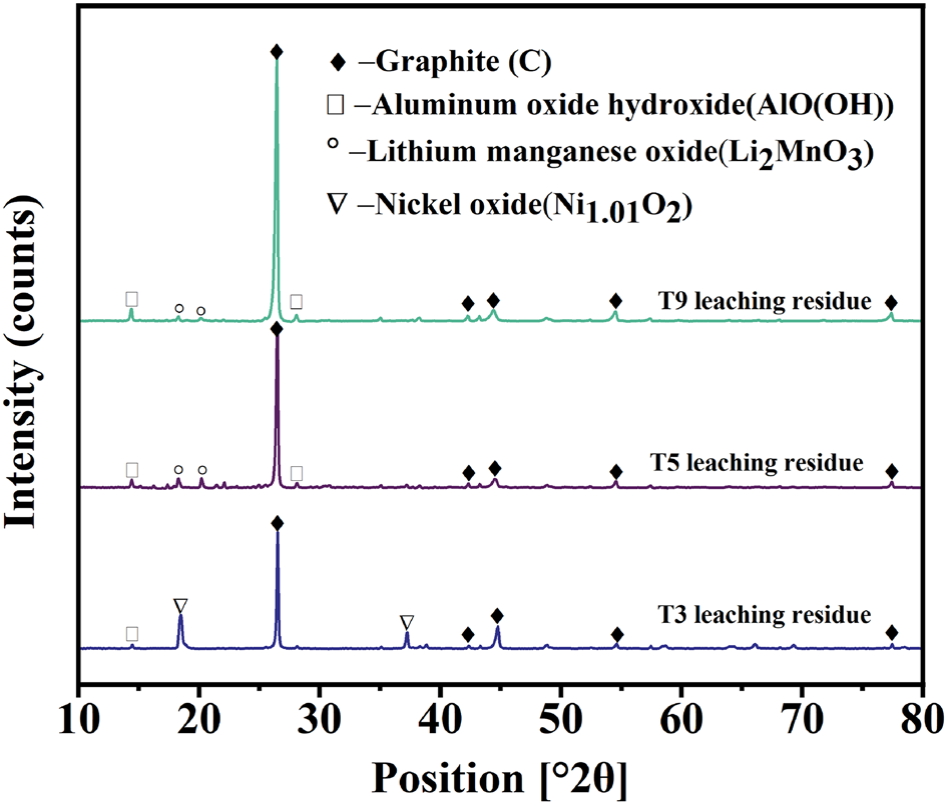
XRD diffractogram of black mass and leaching residues of T3,T5, and T9.

The final concentrations of P and Fe (Supplementary Fig. S6) were 1.3 g/L and 5.2 g/L in T3, 12.0 g/L and 20.9 g/L in T5, and 11.9 g/L and 20.5 g/L in T9. The XRD diffractogram of leach residues in T3, T5, and T9 (Fig. 9) demonstrated that LFP dissolved in these leaching conditions and neither LiFePO_4_ phase nor iron phosphate precipitates were found in the leach residues.

However, Li_2_MnO_3_ (T5 and T9) was found in the leach residue analysis, as well as Ni_1.01_O_2_ in T3 (Fig. 9), which may be explained by the dissolution mechanism related to the structural and chemical evolutions of NMC111. Firstly, the surface of cathode particles (LiNi_1/3_^II^Mn_1/3_^IV^Co_1/3_^III^O_2_)_s_ dissolves by lithium deintercalation and the charge compensation of transition metals, which depend on the proton concentration initiated by an excess of positive charge at the solid/liquid interface of the particles^41^. Consequently, the bulk of the NMC111 cathode particles (LiNi_1/3_^II^Mn_1/3_^IV^Co_1/3_^III^O_2_)_b_ can react with the reductant present in the solution, i.e., with the Fe^2+^ dissolved from LFP. Additionally, dissolved Mn^2+^ or another reductant (H_2_O_2_ in T9 and T10) can reduce LiNi_1/3_^II^Mn_1/3_^IV^Co_1/3_^III^O_2_, leading to a surface reorganization from a layered structure to the metastable birnessite phase Li_2_MnO_3_ (Fig. 9), lowering the leaching efficiency of Mn. In the investigated industrial black mass Li_0.75_Ni_1.05_O_2_ was also analyzed (Fig. 4), with the valence of Ni varying in the range of + 2 to + 4, similar to the study of Billy^41^. It is expected that with a smaller amount of reductive power (LFP) in the system, not all Ni oxides could be reduced, as Ni_1.01_O_2_ was present in the leaching residue of experiment T3 (Fig. 9).

## Discussion

In the exploratory leaching tests, synthetic LFP exhibited high dissolution in acid solutions, reducing synthetic NMC111 effectively and resulting in a significant increase in the extraction of battery metals. Moreover, the concentration of acid was shown to be a crucial factor in the leaching process of synthetic NMC111 with addition of LFP. In the leaching phase of industrial black mass, the incorporation of synthetic LFP as a reductant also contributed positively to metal extraction efficiency. This effect was pronounced with greater addition of LFP, which not only enhanced leaching but also increased acid consumption, consequently lowering the redox potential due to the increased presence of Fe^2+^.

In the present study, the H_2_SO_4_ concentration was limited to a maximum of 2 M, and the temperature was limited to 60 °C, coupled with an agitation speed of 300 rpm. These constraints for their part impose a restriction on extraction efficiency. Higher levels of leaching extraction could potentially be achieved at higher acidities (3–6 M) or elevated temperatures up to 80 °C or 90 °C while maintaining the same ratio of added LFP and black mass. However, increased temperature and acid concentration would also lead to higher operational costs in industrial processing. In addition, due to the lower pH value, the consumption of neutralizing agents in the subsequent purification of pregnant leach solution would be likely. The choice of acidity and temperature depends ultimately on the techno-economic and environmental impacts of holistic processing and is difficult to determine based on an individual unit process.

With LFP batteries expected to increase significantly in the EV market, there will be growing amounts of waste streams containing LFP chemistries in future. If these LFP chemistries were used to reduce other battery waste (NMC and LCO)^35,36^, it would potentially decrease the need for additional reductants in the recycling process. Furthermore, the use of LFP may facilitate selective iron and aluminum removal as iron phosphate, and has also been shown to prevent co-precipitation and losses of valuable transition metals and Li at pH 2.5–4, with improved cake filterability^39^.

Nevertheless, the current study has some limitations. First, the purity of LFP chemistries obtained from spent LIBs is not as high as that of the synthetic LFP employed in the current investigation. This discrepancy in purity could potentially impact the efficiency of metal extraction. Second, it should be noted that NMC-based LIBs encompass not only NMC111 but also variants such as NMC523, NMC622, and NMC811. However, the leaching performance for the remaining NMC types in conjunction with LFP is reported to be comparable to that observed with NMC111.

## Conclusion

This study demonstrates that lithium iron phosphate (LFP) cathode material can serve as an effective reductant for both synthetic NMC111 and real industrial black mass, leading to high extraction levels of transition metals and Li into solution. The process involves the dissolution of LFP in an acid solution, resulting in the release of divalent iron (Fe^2+^), which subsequently undergoes an oxidation reaction by NMC111 or the active cathode material within the black mass, dissolving transition metals and oxidizing Fe^2+^ into trivalent iron (Fe^3+^). The battery metal leaching yields were further improved by increasing the acidity and temperature. In the presence of a 0.9 molar ratio between synthetic LFP and synthetic NMC111, the extraction efficiencies for Ni, Co, Mn, and Li were found to be 86.9%, 100%, 92.5%, and 100%, respectively (2 M H_2_SO_4_, 30 °C, *t* = 2 h). When 28.3 g of synthetic LFP was used as reductant for 50 g of industrial black mass (2 M H_2_SO_4_, 60 °C, *t* = 3 h), the metal extraction percentages were of a similar magnitude: 87.6% Ni, 100% Co, 91.1% Mn, 100% Li, 95.0% Cu, 100% Fe, and 46.3% Al. Early addition of LFP to the leaching system was shown to be beneficial for the final metal extraction, although further use of H_2_O_2_ as a supportive reductant provided only minor improvements in the final recoveries. The suggested leaching approach can support the recycling of first-generation LFP waste that ends up in recycling plants, while facilitating NMC-dominated battery waste recycling via reduction. The use of LFP provides increased Li concentrations for recovery and phosphate ions into solution, which would enhance further iron and aluminum removal.

### Supplementary Information

Supplementary Figures.

## Data Availability

The datasets generated during and/or analyzed during the current study are available from the corresponding author on reasonable request.
