# Supplementary material for: Leaching of NMC industrial black mass in the presence of LFP
Source: Sci Rep. 2024 May 11;14:10818. doi: 10.1038/s41598-024-61569-3 (PMC11088664; doi:10.1038/s41598-024-61569-3)
Supplement: Supplementary file 1 — Supplementary Figures. [file 41598_2024_61569_MOESM1_ESM.docx]

**Leaching of NMC industrial black mass in the presence of LFP**

Yuanmin Zou^a^, Alexander Chernyaev^a,b^, Ossama Muhammad ^a^, Sipi Seisko^a^, Mari Lundström^a*^

^a^ Department of Chemical and Metallurgical Engineering, School of Chemical Engineering, Aalto University, 00076 Aalto, Finland

^b^ Metso Outotec Research Center, Kuparitie 10, 28101 Pori, Finland

*Corresponding author: [mari.lundstrom@aalto.fi](mailto:mari.lundstrom@aalto.fi)

**
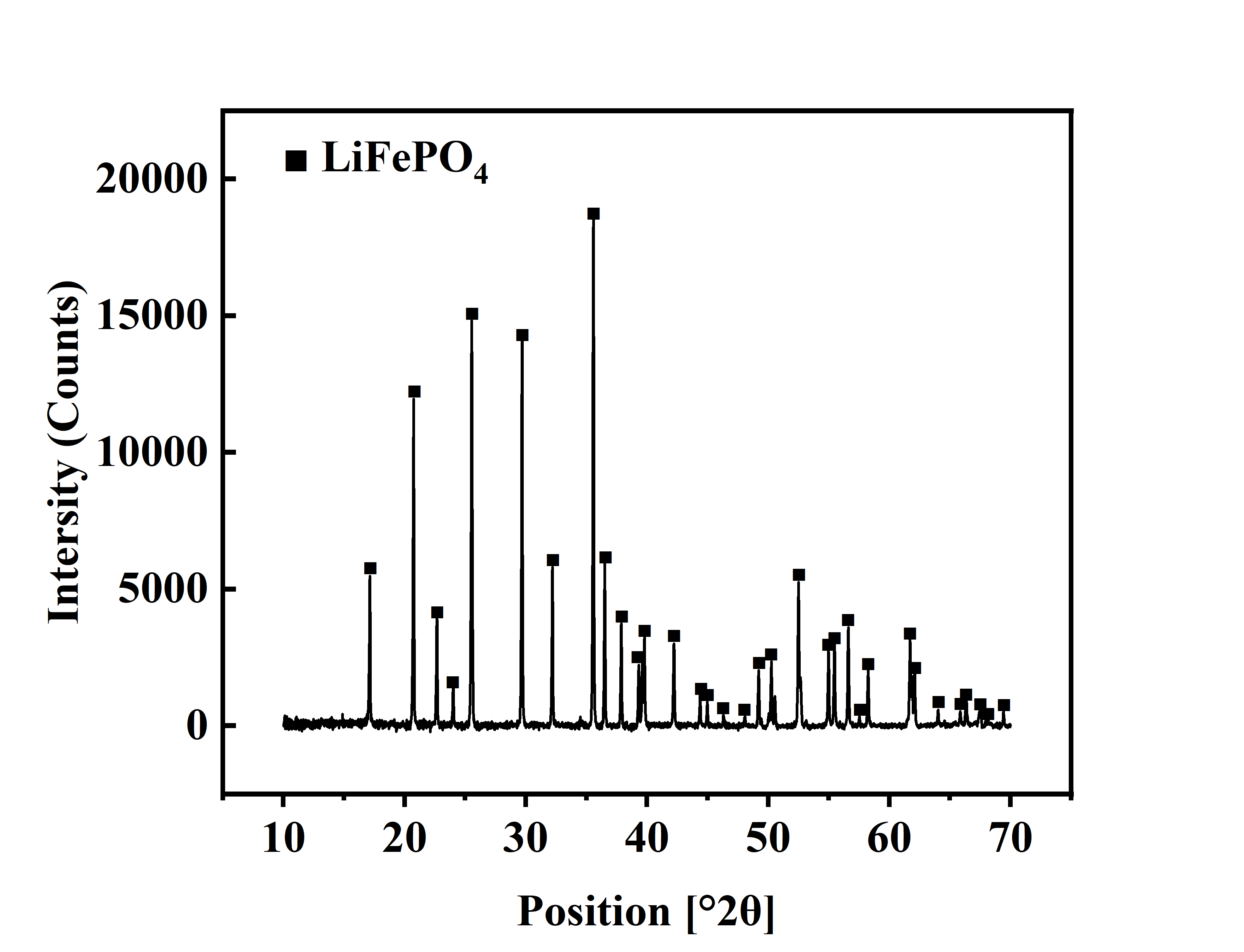
**

**Figure S1.** XRD diffractogram of synthetic LFP

**
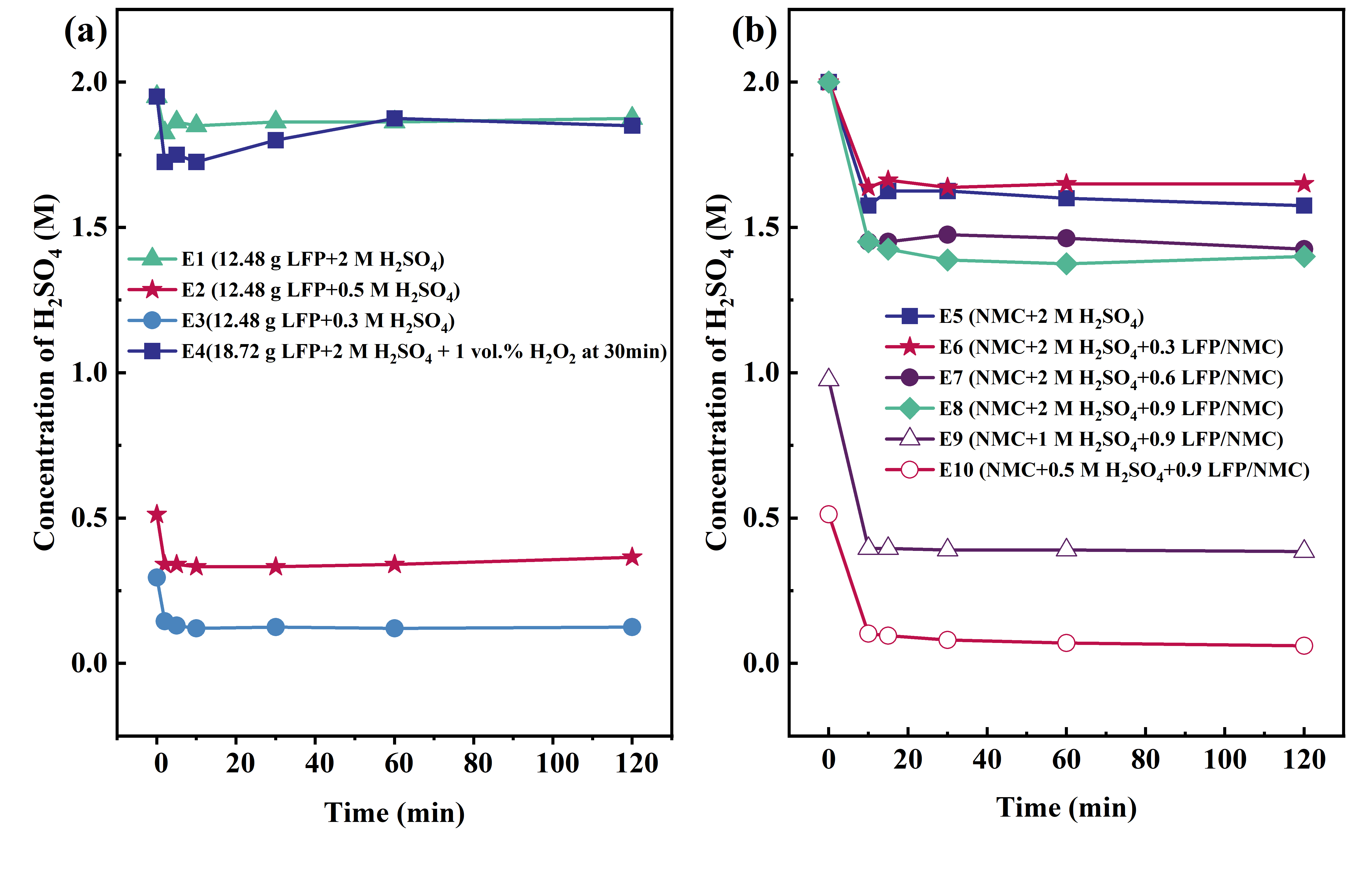
** Figure S2. Acidity (a) E1-E4 and (b) E5-E10.


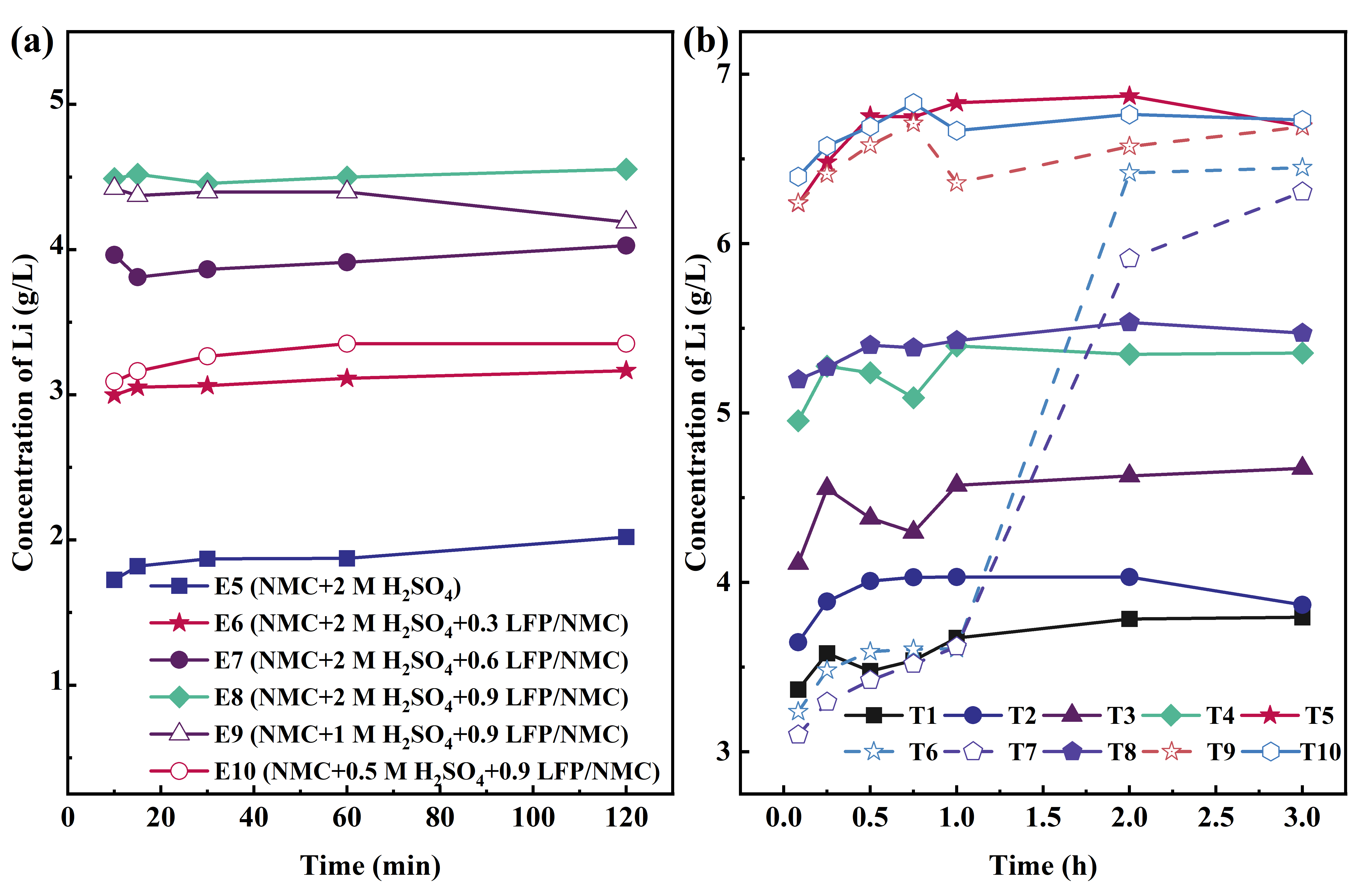


Figure S3. Concentration of Li (a)in E5-E10 and (b) in T1-T10


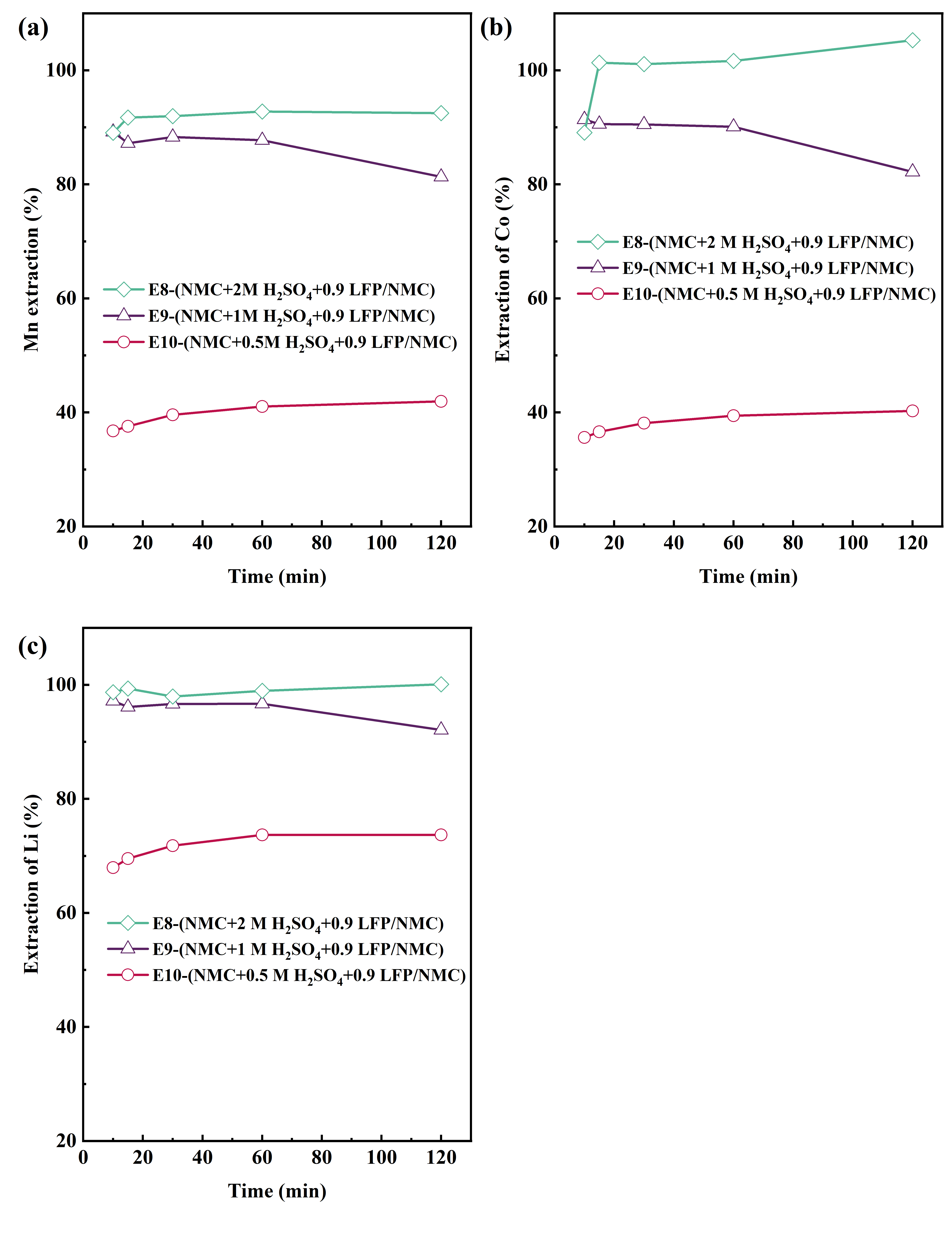
Figure S4. (a) Mn, (b) Co, and (c) Li leaching yield at different H_2_SO_4_ concentrations.

Figure S5 shows the redox potential recorded in the leaching tests. During the first hour, there was an increase in redox potential across all tests, except for T8. The total rise in redox potential from beginning to end was 280 mV in T1, 368 mV in T2, 300 mV in T3, 286 mV in T4, and 285 mV in T5, vs. Ag/AgCl. After the first hour, the redox potential remained relatively stable for all these tests. The final redox potential values for T1 to T4 fell within the range of 960 – 1000 mV vs. Ag/AgCl. However, T5 stood out with the lowest value, remaining at 740 mV vs. Ag/AgCl. This implies that the introduction of a higher quantity of LFP resulted in a decreased redox potential along with a greater generation of Fe^2+^. Out of experiments T5 (2.0 M acid), and T8 (1.0 M acid), T5 consistently maintained the highest redox potential throughout the entire reaction period, while T8 consistently had the lowest. Ultimately, the final redox potential was 693 mV vs. Ag/AgCl in T11 and 261 mV vs. Ag/AgCl in T8, both of which were lower than that observed in T5. The results suggest that in this experimental series, a decrease in acidity is associated with a lowered redox potential in the leach solution. This is because, at lower acidity levels, not all of the Fe^2+^ reacts adequately with the available acid, resulting in a significant amount of dissolution. In scenarios where there is an abundance of Fe^2+^ and lower concentrations of other metals, the redox potential tends to be lower but more stable. Conversely, higher acidity levels typically correspond to elevated redox potential levels.


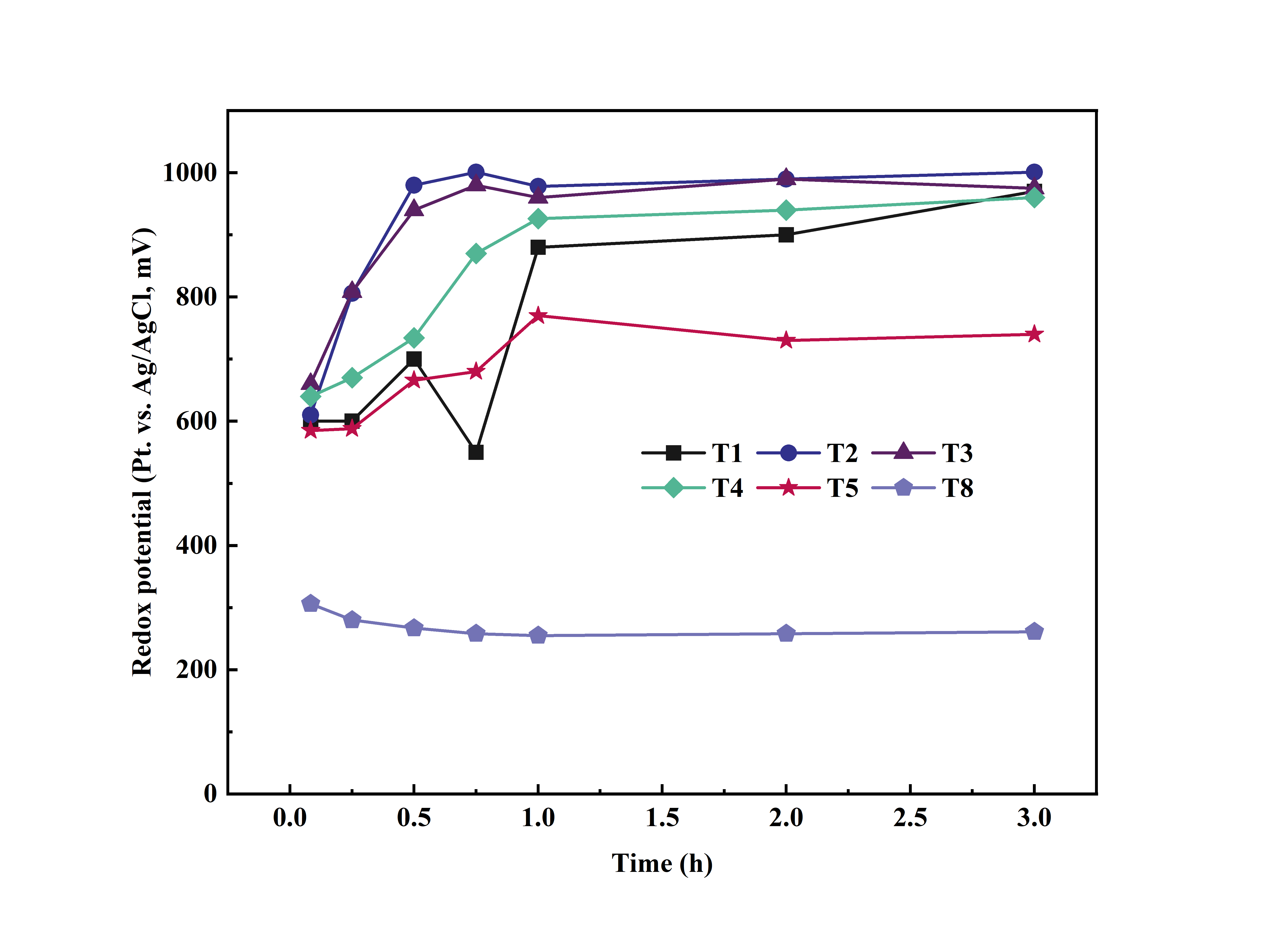


Figure S5. Redox potential in experiments T1 – T5 and T8.


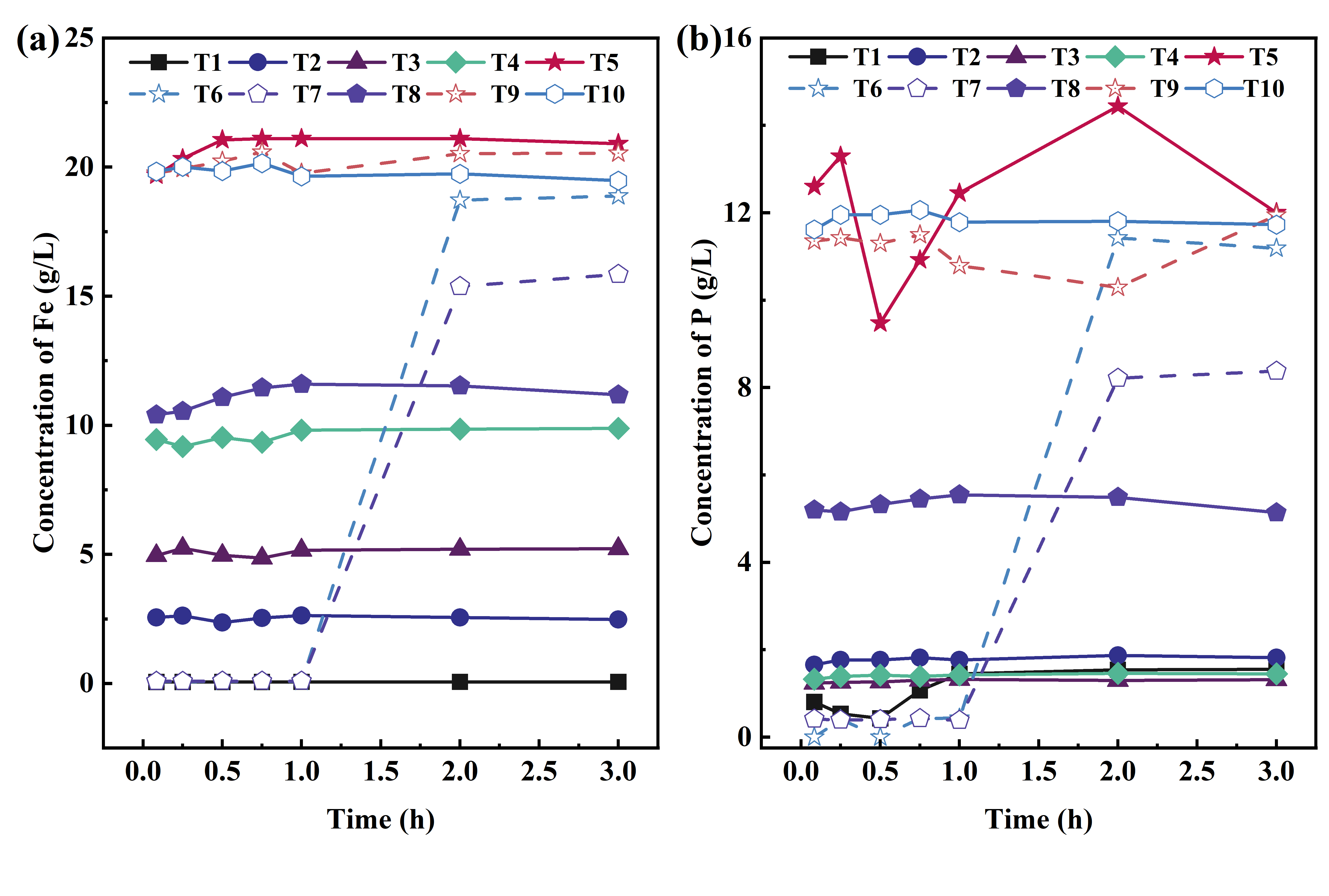


Figure S6. Concentration in T1-T10 for (a)Fe and (b) P
